# Supplementary material for: MARTApp: software for the processing and reconstruction of synchrotron-radiation-based magnetic tomographies
Source: J Synchrotron Radiat. 2025 Jun 26;32(Pt 4):1095–104. doi: 10.1107/S1600577525004485 (PMC12236238; doi:10.1107/S1600577525004485)
Supplement: Supplementary file 1 [file s-32-01095-sup1.pdf]

JOURNAL OF SYNCHROTRON RADIATION

Volume 32 (2025)

Supporting information for article:

MARTApp: software for the processing and reconstruction of synchrotron-radiation-based magnetic tomographies

A. Estela Herguedas-Alonso, Joaquín Gómez Sánchez, Claudia Fernández-González, Andrea Sorrentino, Salvador Ferrer, Eva Pereiro and Aurelio Hierro-Rodriguez

Supporting Information: MARTApp, software for the processing and reconstruction of synchrotron radiation-based magnetic tomographies.

A.E. Herguedas-Alonso, J. Gómez Sánchez, C. Fernández-González, A. Sorrentino, S. Ferrer, E. Pereiro and A. Hierro-Rodriguez.

Downgrade of the resolution due to artifacts.

In Magnetic Volume Tomography (MVT), several factors can degrade the resolution of the reconstruction, such as the limited rotation angle (±65°), image noise, and alignment inaccuracies. To assess these effects, we have performed different reconstructions and obtained their resolution. For visualization purposes, we have only reconstructed the absorption, as the resolution for the magnetization is similar. We have created a 3D Shepp-Logan phantom in a space of 512x512x250 with 2 nm voxel size. Transmission images were then computed from the volume. These images were obtained by using the Lambert-Beer law to calculate the intensity at each pixel of the detector for tilt angles spanning from -90° to 90° with an angular step of 2°. Since no magnetic signal was introduced in our phantom, only one tilt series is required in the reconstructions. The reconstructions have been performed using the algorithm in [1].

The axial resolution has been evaluated by comparing the reconstruction with the ground truth. The lateral resolution can be obtained similarly but it is not shown here since the geometry of the microscope allows to resolve features on the imaging plane (XY) with higher resolution compared to the depth (Z axis). To compute the resolution, first we have extracted the same profile along the Z axis from both volumes, where the intensity or absorbance decays to zero as shown in Fig. S1(a) in red. The profiles are presented in Fig. S1(b) in blue and red for the ground truth and the reconstruction respectively. The reconstruction is the result of a convolution between the true configuration of the magnetization and the transmission function of the microscope. This function is simulated as a normalized Gaussian function:

$$G(x) = \frac{1}{\sigma\sqrt{2\pi}} \exp\left(\frac{-x^2}{2\sigma^2}\right)$$

where σ is half of the full-width-half-maximum value (FWHM), which introduces the optical resolution of the system. Therefore, it is found the parameter σ that minimizes the difference between the reconstruction and the convolution between the ground truth and the Gaussian function. The Gaussian function for the profile of Fig. S1(a) is depicted in Fig. S1(c), resulting in a FWHM and an axial resolution of 11.8 nm. The convolution between the Gaussian and the ground truth is shown in green in Fig. S1(b).

To introduce the artifacts in the reconstructions and observe the change in the resolution, we have performed 4 reconstructions where the images were modified as follows (each reconstruction includes the artifacts from the previous reconstructions):

1. Using the raw images of the dataset, whose resolution is shown in Fig. S1.
2. Replicating the conditions of image acquisition under a real detector, i.e. applying a Gaussian filter to mimic the optical resolution of the microscope (30 nm), subsampling

to 10 nm and adding Poisson noise with 20000 photons of amplitude, in agreement with the flux for 1 s at 520 eV in MISTRAL beamline [2,3].

3. Setting the maximum angular range of the images to ±65° to have a realistic missing wedge.

4. Introducing random misalignment of maximum 2 pixels (20 nm) for each image.

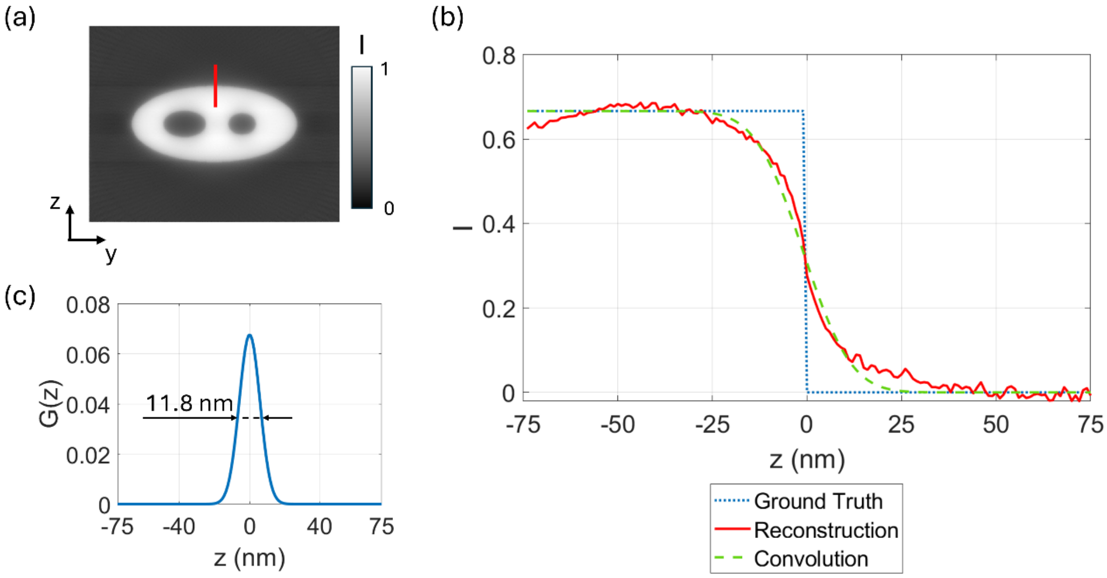

Figure S1. (a) Image from the reconstruction of the phantom. (b) Profiles taken from the ground truth (blue), reconstruction (red) and the convolution of the ground truth with the Gaussian function depicted in (c) (green).

The values of the axial resolution obtained from the same profiles are shown in Table S1. As expected, the value of the resolution is lower for the raw data. The biggest downgrade of the resolution is when limiting the angular range with a resolution of 65 nm. Since the reconstruction algorithm creates the 3D volume from 2D slices in the Fourier space, the missing frequencies introduce blurring and distorted features along the Z axis, reducing its resolution.

Artifact	1<br>Raw data	2<br>Microscope transmission function	3<br>Missing wedge	4<br>Misalignment
Resolution (nm)	11.8	14.24	64.54	69.87

Table S1. Values of the resolution for the reconstructions including the artifacts.

Performance and scalability.

To illustrate the computational performance of the algorithms implemented in MARTApp, we present here the runtime for both the image alignment and reconstruction using the phantom dataset described earlier. As in the previous section, the transmittance images are computed from a single tilt series, with 91 projections, distributed from -90° to +90° and angular step of 2°. Four datasets are calculated, where the sizes of the images are 256x256, 512x512, 1024x1024,

and 2048x2048. Each dataset has been computed from a phantom volume size according to the image size previously mentioned.

To study the potential of the alignment algorithms, random misalignments were introduced in the dataset, with a maximum displacement of 30 pixels. For example, in the case of an image size of 256×256 pixels, the maximum displacement introduced was 12 pixels. Fig.S2(a) and Table S2 shows the time required for two types of image alignment processes: in blue, alignment between polarizations C+ and C- for the complete tilt series (paralellized); in red, the tilt alignment of all 91 projections. In both alignments the PyStackReg algorithm [4] was used. As expected, the alignment time increases with image size. The alignment was performed on a computer Intel Core i5 (3.10 GHz) CPU with 6 cores.

Image size (pixels)	256x256	512x512	1024x1024	2048x2048
Alignment between pair of images Time (s)	5.83	10.76	30.31	152.57
Tilt alignment Time (s)	4.48	18.00	79.80	378.45

Table S2. Runtime of the alignments depending on the image sizes.

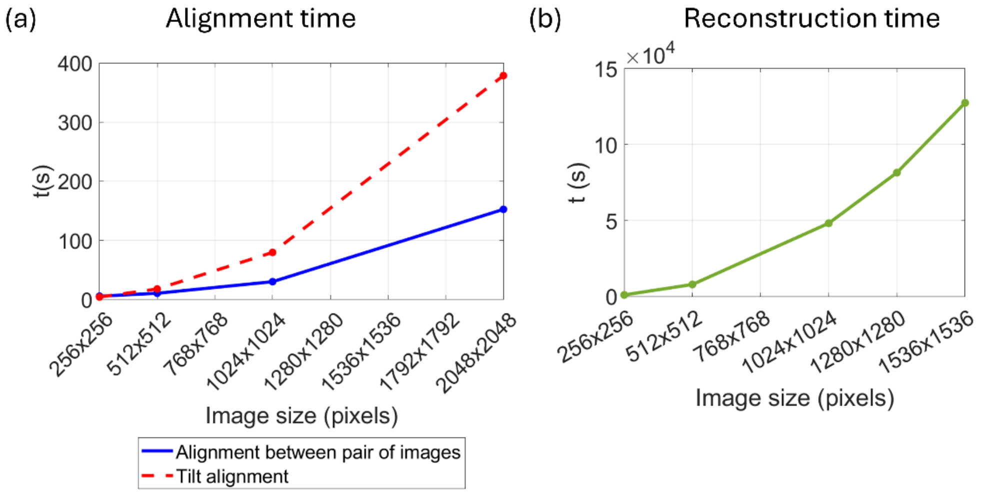

Figure S2. Runtimes of MARTApp for (a) the alignment between C+ and C- (in blue) and the tilt alignment (in red) and (b) reconstruction times for dataset with one tilt series with 91 projections with different image sizes.

For the reconstructions the algorithm cited in [1] with 10 iterations of SIRT was used. The X and Y dimensions of the reconstructed volume are equal to the image size while the Z dimension has been fixed at 250 pixels for all reconstructions. Fig. S2(b) and Table S3 present the reconstruction time for different image sizes. The reconstruction time scales exponentially with image size. For the smallest image size (256x256 pixels), reconstruction took approximately 17 min and 55 s, while for the largest size (1536x1536 pixels), it increased to over 35 hours. For larger volume dimensions, the reconstruction process could not be completed due to insufficient system memory (RAM). An Intel Xeon CPU (3.70 GHz) with 128 GB RAM was employed to perform the reconstructions.

Image size (pixels)	256x256	512x512	1024x1024	1280x1280	1536x1536
Voxels ($x10^6$)	16.384	65.536	262.144	409.600	589.824
Time (s) ($x10^4$)	0.1075	0.7950	4.8244	8.1541	12.7472

Table S3. Runtime of the reconstruction algorithm for the different image and volume sizes.

References

[1] Hierro-Rodriguez, Aurelio, et al. "3D reconstruction of magnetization from dichroic soft X-ray transmission tomography." *Synchrotron Radiation* 25.4 (2018): 1144-1152.

[2] Otón, Joaquín, et al. "Measurement of the modulation transfer function of an X-ray microscope based on multiple Fourier orders analysis of a Siemens star." Optics express 23.8 (2015): 9567-9572.

[3] Sorrentino, Andrea, et al. "MISTRAL: a transmission soft X-ray microscopy beamline for cryo nano-tomography of biological samples and magnetic domains imaging." *Synchrotron Radiation* 22.4 (2015): 1112-1117.

[4] Thevenaz, Philippe, Urs E. Ruttimann, and Michael Unser. "A pyramid approach to subpixel registration based on intensity." *IEEE transactions on image processing* 7.1 (1998): 27-41.
